# Acute Spinal Cord Infarction Secondary to Spinal Dural Arteriovenous Fistula: A Rare Cause of Reversible Paraplegia

**DOI:** 10.7759/cureus.83135

**Published:** 2025-04-28

**Authors:** Chien-Wei Li, Chun-Hsin Teng, Tzu-Chao Lin, I-Hsiao Yang, Poyin Huang

**Affiliations:** 1 Department of Neurology, Kaohsiung Medical University Hospital, Kaohsiung, TWN; 2 Department of Neurology, Kaohsiung Municipal Min-Sheng Hospital, Kaohsiung, TWN; 3 Department of Neurology, Kaohsiung Municipal Siaogang Hospital, Kaohsiung, TWN; 4 Department of Radiology, Kaohsiung Medical University Hospital, Kaohsiung, TWN

**Keywords:** acute paraplegia, dural arteriovenous fistula, embolization, neurogenic bladder, spinal cord infarction

## Abstract

Acute spinal cord infarction due to spinal dural arteriovenous (AV) fistula is a rare etiology of acute paraplegia that challenges timely diagnosis. We report a case of a 76-year-old male presenting with sudden bilateral lower extremity weakness, urinary retention, and constipation. Diagnostic evaluation revealed an AV fistula causing spinal cord infarction, confirmed by imaging. Urgent embolization resulted in significant recovery of muscle strength, though neurogenic bladder persisted. This case underscores the importance of identifying reversible causes of acute paraplegia and the critical role of prompt intervention.

## Introduction

Acute paraplegia is a medical emergency requiring timely diagnosis to address reversible etiologies and prevent permanent neurologic damage. Common non-traumatic causes include Guillain-Barré syndrome (GBS), transverse myelitis, and tumor-related compression or metastasis, often accompanied by autonomic dysfunction. Rarely, spinal cord infarction secondary to vascular malformations, such as spinal dural arteriovenous (AV) fistulas, may present similarly [1]. Though uncommon, early recognition and intervention can significantly improve the outcome. Here, we report a case of acute paraplegia resulting from AV fistula-induced spinal cord infarction, emphasizing the critical importance of timely recognition and early intervention.

## Case presentation

A previously healthy 76-year-old male presented with acute bilateral lower extremity soreness below the umbilicus and lower back pain. Three days later, he developed urinary retention and constipation, followed by sudden bilateral lower extremity weakness with an ascending pattern on day 4. Additionally, he described a tingling sensation, initially in the right leg and then the left. No preceding trauma, infection, vaccination, or systemic symptoms were reported. Initial differential diagnoses included GBS, transverse myelitis, and anterior spinal artery infarction.

**Figure 1:**
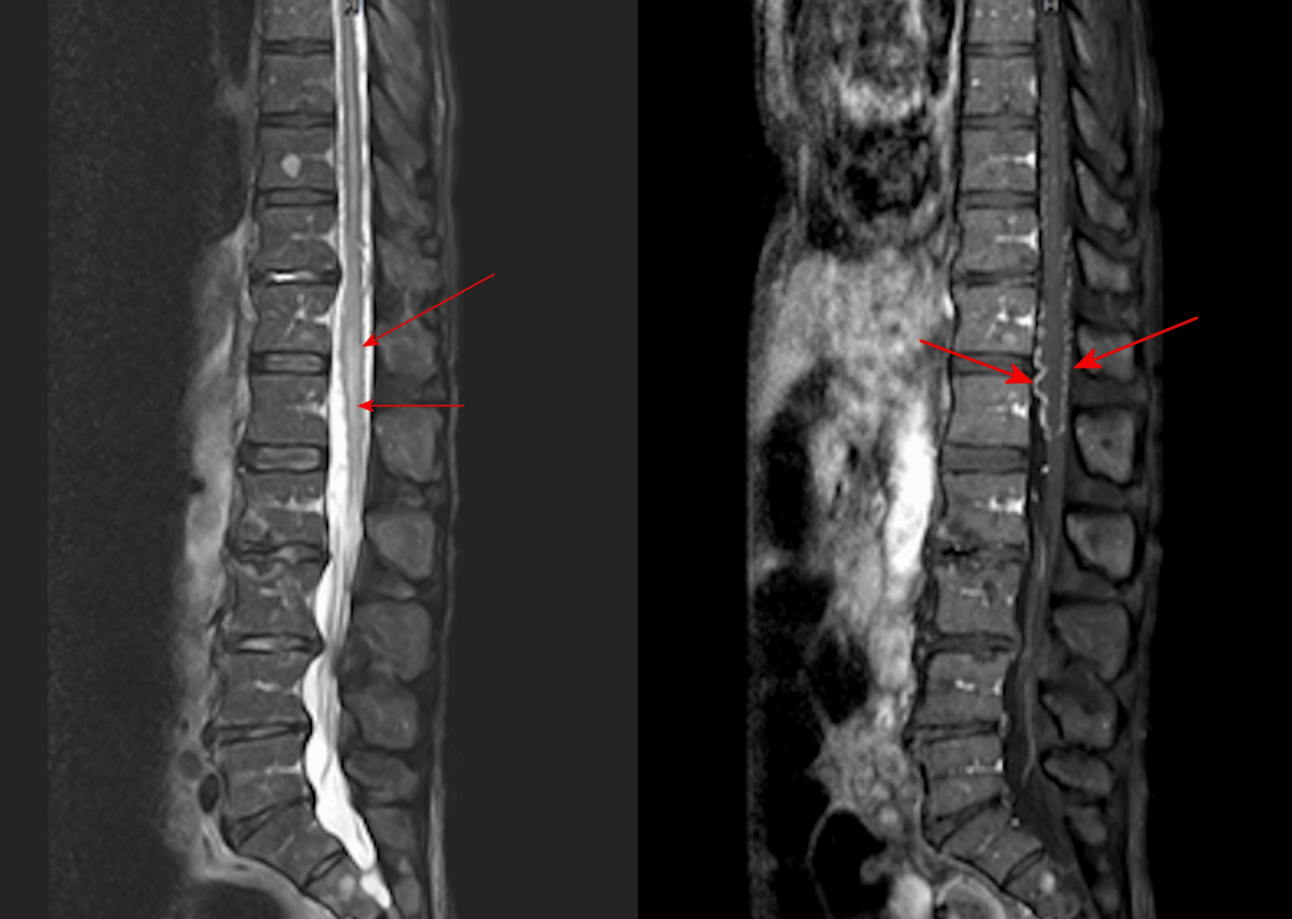
Myelopathy at T12-L1 under thoracolumbar spine magnetic resonance imaging (MRI) Coronal T2-weighted MRI of the thoracolumbar spine showing longitudinal spinal cord edema (red arrows), suggesting acute infarction due to venous congestion resulting from a dural arteriovenous fistula.

**Figure 2:**
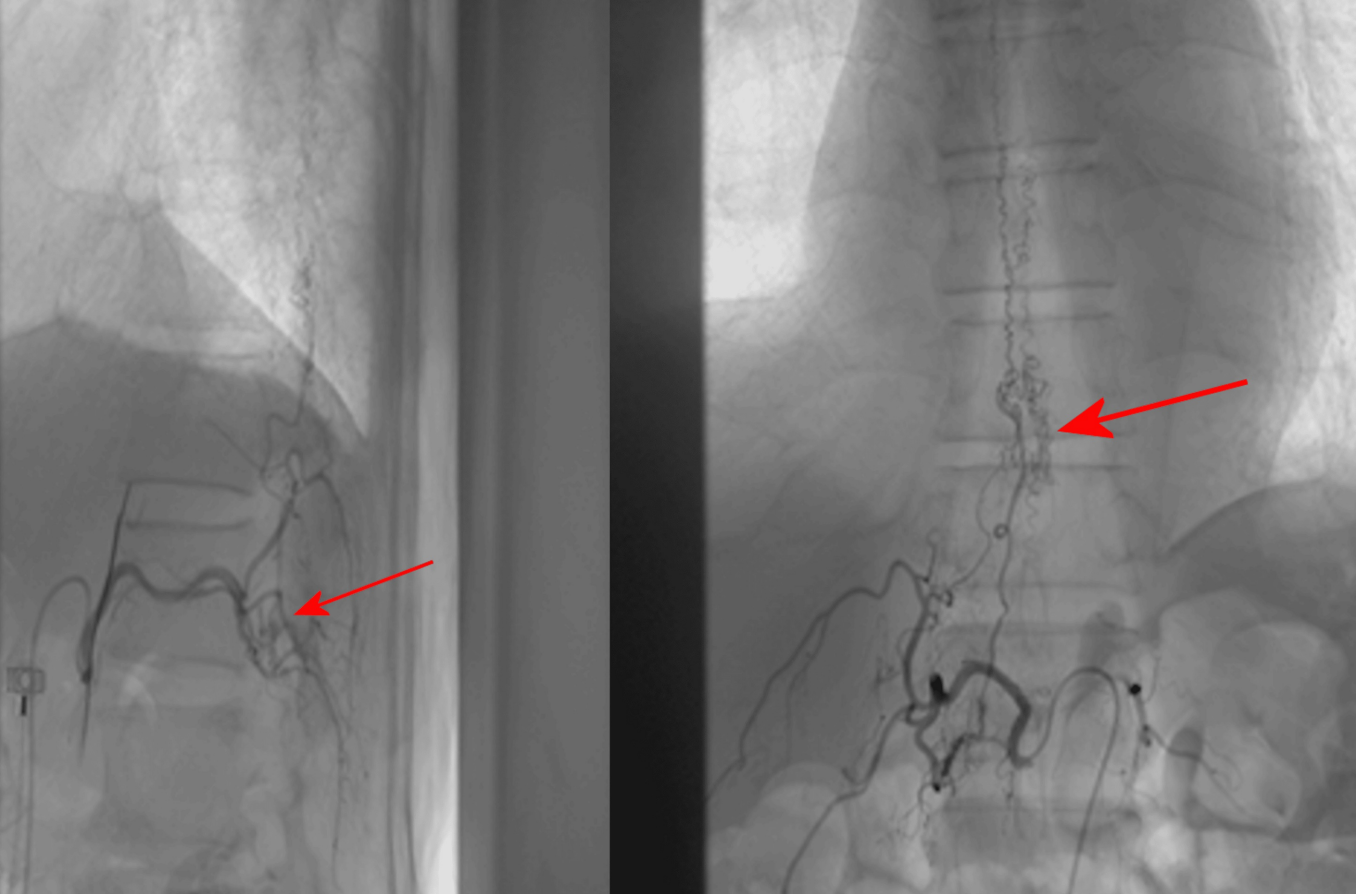
An AV fistula at T12-L1 with venous congestion under angiography Digital subtraction angiography demonstrating an AV fistula at T12-L1 (red arrow) with associated venous congestion (large red arrow), thereby confirming the underlying cause of the spinal cord infarction. AV: arteriovenous

On admission, neurologic examination revealed flaccid weakness in both lower extremities (worse distally), bilateral plantarflexion, impaired joint position sense, and relatively preserved pinprick sensation. Pulse therapy of corticosteroids was used initially but in vain, and muscle strength deteriorated by day 2 to a Medical Research Council (MRC) scale score of 1 proximally and 0 distally bilaterally. Thoracolumbar spine magnetic resonance imaging (MRI) with and without contrast demonstrated myelopathy at T12-L1, suggestive of acute spinal cord infarction secondary to an AV fistula (Figure 1). Further digital subtraction angiography confirmed the diagnosis of AV fistula at T12-L1 with venous congestion (Figure 2).

Neurosurgical consultation deemed surgery inappropriate, and urgent embolization was performed. After the therapeutic procedure, his strength in bilateral lower extremities improved markedly, and paresthesia partially resolved. However, the neurogenic bladder persisted, necessitating an indwelling catheter. He was transferred to a rehabilitation unit for ongoing care.

## Discussion

Acute paraplegia requires urgent assessment, since delay in diagnosis may result in irreversible neurological deficits. In comparison with other frequent culprits, spinal cord infarction is extremely rare, accounting for less than 1% of all infarctions [2]. The resulting sequelae, including paraplegia, quadriplegia, ataxia, or autonomic dysfunction, are determined by the spinal segment involved. In most cases, spinal cord infarction stems from extravertebral artery or aortic injury (e.g., surgical clamping or severe hypotension), while spinal vascular malformations (e.g., dural AV fistulas) are only implicated in a minority of instances. Representing 70% of spinal vascular malformations, dural AV fistulas cause infarction via venous congestion, leading to intramedullary edema and hypoxia [3,4].

In this patient, thoracolumbar MRI helped with identifying spinal infarction, and angiography further confirmed a dural AV fistula as the cause. Embolization rapidly improved motor function, consistent with prior reports of reversible deficits when venous hypertension is alleviated promptly. However, autonomic dysfunction, such as neurogenic bladder, often persists, reflecting differential recovery potential among spinal cord functions [5].

## Conclusions

This case illustrates that spinal cord infarction secondary to dural AV fistula, though rare, should be considered in case of acute paraplegia with autonomic features. Urgent MRI and angiography, coupled with timely embolization, can reverse motor deficits and avert catastrophic outcomes. Clinicians should remain vigilant and consider these reversible causes in their differential diagnosis.
